# HbA_1c_ Performance in African Descent Populations in the United States With Normal Glucose Tolerance, Prediabetes, or Diabetes: A Scoping Review

**DOI:** 10.5888/pcd18.200365

**Published:** 2021-03-11

**Authors:** Lakshay Khosla, Sonali Bhat, Lee Ann Fullington, Margrethe F. Horlyck-Romanovsky

**Affiliations:** 1 Department of Health and Nutrition Sciences, Brooklyn College, City University of New York, Brooklyn, New York; 2 College of Medicine, SUNY Downstate Health Sciences University, Brooklyn, New York; 3 Library Department, Brooklyn College, City University of New York, Brooklyn, New York; 4 Center for Systems and Community Design, Graduate School of Public Health and Health Policy, City University of New York, New York, New York

## Abstract

**Introduction:**

African descent populations in the United States have high rates of type 2 diabetes and are incorrectly represented as a single group. Current glycated hemoglobin A_1c_ (HbA_1c_) cutoffs (5.7% to <6.5% for prediabetes; ≥6.5% for type 2 diabetes) may perform suboptimally in evaluating glycemic status among African descent groups. We conducted a scoping review of US-based evidence documenting HbA_1c_ performance to assess glycemic status among African American, Afro-Caribbean, and African people.

**Methods:**

A PubMed, Scopus, and Cumulative Index to Nursing and Allied Health Literature (CINAHL) search (January 2020) yielded 3,238 articles published from January 2000 through January 2020. After review of titles, abstracts, and full texts, 12 met our criteria. HbA_1c_ results were compared with other ethnic groups or validated against the oral glucose tolerance test (OGTT), fasting plasma glucose (FPG), or previous diagnosis. We classified study results by the risk of false positives and risk of false negatives in assessing glycemic status.

**Results:**

In 5 studies of African American people, the HbA_1c_ test increased risk of false positives compared with White populations, regardless of glycemic status. Three studies of African Americans found that HbA_1c_ of 5.7% to less than 6.5% or HbA_1c_ of 6.5% or higher generally increased risk of overdiagnosis compared with OGTT or previous diagnosis. In one study of Afro-Caribbean people, HbA_1c_ of 6.5% or higher detected fewer type 2 diabetes cases because of a greater risk of false negatives. Compared with OGTT, HbA_1c_ tests in 4 studies of Africans found that HbA_1c_ of 5.7% to less than 6.5% or HbA_1c_ of 6.5% or higher leads to underdiagnosis.

**Conclusion:**

HbA_1c_ criteria inadequately characterizes glycemic status among heterogeneous African descent populations. Research is needed to determine optimal HbA_1c_ cutoffs or other test strategies that account for risk profiles unique to African American, Afro-Caribbean, and African people living in the United States.

**Summary**

**What is already known on this topic?**

Type 2 diabetes disproportionately affects African descent groups, yet contributing factors are often overlooked. Studies show that glycated hemoglobin A_1c_ (HbA_1c_) underperforms as a screening and diagnostic tool among ethnic cohorts of this population.

**What is added by this report?**

This review demonstrates that current HbA_1c_ cutoffs overestimate glycemic status in African Americans and underestimate glycemic status in Afro-Caribbeans and Africans. It identifies gaps in the scientific literature, especially among Afro-Caribbeans.

**What are the implications for public health practice?**

Type 2 diabetes screening and diagnostic tests must account for genetic, biochemical, and socioeconomic factors. To ensure early type 2 diabetes detection, heterogeneity within African descent groups must be recognized, and more reliable testing strategies must be identified.

## Introduction

People of African descent in the United States have a disproportionate burden of type 2 diabetes; prevalence is higher in African descent populations, 14%, compared with White populations of European descent (White populations), 9% (1). Additionally, African descent populations are represented as a single group, despite being comprised of African American (91%), Afro-Caribbean (4.7%), and African (3.7%) people (2,3). Limited evidence examines how intraethnic differences in cardiometabolic risk criteria, social determinants of health, and genetic admixture affect diabetes risk in these 3 populations (4,5). Current glycated hemoglobin A_1c_ (HbA_1c_) cutoffs (HbA_1c_ 5.7% to less than 6.5% for prediabetes; HbA_1c_ of 6.5% or higher for type 2 diabetes), determined from predominantly White population cohorts (4–8), may perform suboptimally in evaluating glycemic status in this diverse population of African American, Afro-Caribbean, and African populations (9–12). African American people may have higher HbA_1c_ values across the glycemic spectrum (9,13), and African immigrants may have lower HbA_1c_ values compared with White people (14). To ensure accurate detection of type 2 diabetes, there is a need to understand the ability of HbA_1c_ to correctly classify type 2 diabetes status and to evaluate intraethnic variation among African American, Afro-Caribbean, and African people (15–17).

Compared with random glucose, fasting plasma glucose (FPG), and the 2-hour oral glucose tolerance test (OGTT), HbA_1c_ has multiple benefits. It does not require fasting, tracks plasma glucose over the preceding 2 to 3 months, and better predicts complications such as cardiovascular disease (4,18). The HbA_1c_ test is stable, unaffected by external variables (eg, exercise, recent meals, and environmental stressors), and easily added to blood tests (19,20). However, interpretation of HbA_1c_ results is affected by the reduced lifespan of red blood cells in patients with type 2 diabetes, anemia, and hemoglobinopathies, conditions which disproportionately affect African descent populations (21–25).

The goal of our study was to conduct a scoping review of US-based peer-reviewed evidence documenting HbA_1c_ performance in African American, Afro-Caribbean, and African populations in the United States with the objectives of 1) summarizing evidence on HbA_1c_ performance in each subethnic group; 2) demonstrating variations in HbA_1c_ performance by each subethnic group; and 3) identifying potential future areas of research.

## Methods

### Data sources

In early January 2020, we searched PubMed, Scopus, and Cumulative Index to Nursing and Allied Health Literature (CINAHL) for peer-reviewed studies published between January 1, 2000, and January 1, 2020, by using complex search strings that were tested and developed in partnership with our institution’s health sciences librarian (L.A.F.). The search string included medical subject headings (MeSH) terms and key words such as “African continental ancestry group,” “African Americans,” “Caribbean,” and “West Indian” to describe population groups and “Glycated Hemoglobin A,” “hemoglobin A_1c_,” and “hba_1c_” to describe the testing indicator of interest for type 2 diabetes (Appendix).

### Study selection

Throughout the review process, we screened articles for studies meeting the following inclusion criteria:

1. Articles were original studies published between January 2000 and January 2020, that evaluated HbA_1c_ performance in African descent groups.
2. Study populations included African Americans, Afro-Caribbeans, or Africans.
3. Study participants were living in the United States.
4. Study was a database analysis, epidemiologic study, or clinical study.
5. HbA_1c_ performance was reported specifically in one or more of the African descent groups.
6. HbA_1c_ performance was assessed in healthy populations or for screening or diagnosis of prediabetes or type 2 diabetes.
7. HbA_1c_ performance was assessed by statistical methods (eg, sensitivity, specificity, and positive predictive value), compared with other tests in the same population, or compared African descent populations to other racial groups.

During the study selection process, we included studies that compared various diabetes screening tests against HbA_1c_, including the OGTT, FPG, and glycated protein tests, to avoid excluding major findings. Although the OGTT is considered optimal for comparison, it is far more costly, resource intensive, and time consuming than the FPG and glycated protein tests (6–8); additionally, research supports the use of other tests along with OGTT or in place of OGTT to enhance detection of diabetes (7,18–22). Because African descent populations are less likely to be adequately represented in clinical research and simultaneously experience health care inequities (4,19), we wanted to be inclusive of all the data, in comparison to HbA_1c_, that were available for the populations.

On the basis of the title and abstract review, we excluded articles that did not match the set inclusion criteria above (Figure). Two authors (L.K. and S.B.) conducted independent title and abstract reviews. In the full-text review, we excluded articles with insufficient data (eg, case studies), narrative reviews, and articles that fell under a previously set exclusion criterion not detected during the title and abstract review. Full-text articles for potential studies were reviewed by 2 authors (L.K. and S.B.) independently. When multiple exclusion criteria were met, we categorized the article by the exclusion criterion that appeared first in title, abstract, or full text review. A third author (M.H.R.) verified that the exclusion criteria were relevant throughout the article.

**Figure:**
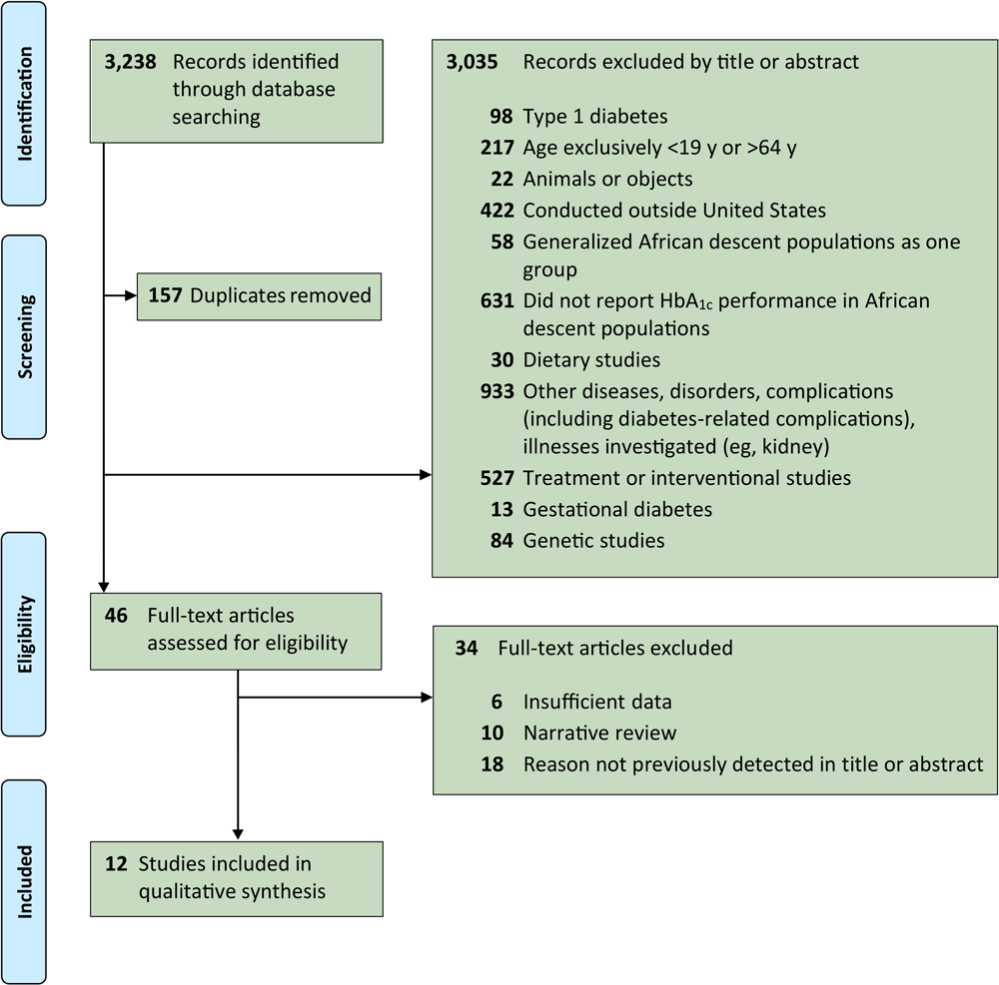
Flow diagram of the study selection process for glycated hemoglobin A_1c_ (HbA_1c_) testing performance in African descent populations in the United States, using PRISMA (Preferred Reporting Items for Systematic Reviews and Meta-Analyses). Studies were published January 1, 2000, to January 1, 2020.

During the identification process, 3,238 records were identified through database searching. In the screening phase, 3,081 records were screened after 157 duplicates were removed. Records were excluded by using a title and abstract review (n = 3,035) by the following exclusion criteria: topic was type 1 diabetes (n = 98); age was exclusively less than 19 years or greater than 64 years (n = 217); topic was animals or objects (n = 22); study was conducted outside the United States (n = 422), study generalized African descent populations as one group (n = 58); study did not report HbA_1c_ performance in African descent populations (n = 631); topic was a dietary study (n = 30); topic was other diseases, disorders, complications (including diabetes-related complications), or illnesses (eg, kidney) (n = 933); topic was a treatment or interventional study (n = 527); topic was gestational diabetes (n = 13); or topic was a genetic study (n = 84). After this screening process, the remaining 46 full-text articles were assessed for eligibility. Of these, 34 full-text articles were excluded based on the following exclusion criteria: insufficient data (n = 6); being a narrative review (n = 10); or for a reason not previously detected in the title or abstract (n = 18). The inclusion stage yielded 12 studies to be included in qualitative synthesis.

### Data extraction

We created a data extraction sheet to record the study author and name, populations (sample size, male/female breakdown, race/ethnicity distribution, age, and study location), HbA_1c_ laboratory methods, study design, HbA_1c_ evaluation methods, findings, and HbA_1c_ performance. We successfully retrieved any missing information by 1) searching through cited articles from which the studies retrieved data; 2) identifying parent studies and protocol descriptions given in prior publications; and 3) emailing corresponding authors. HbA_1c_ performance was classified using 2 labels: 1) greater risk of false positive (GRFP) label indicated that the HbA_1c_ test may result in overdetection of glycemic status (eg, type 2 diabetes) that the study is measuring or 2) greater risk of false negatives (GRFN) label indicated that the HbA_1c_ test may result in underdetection of glycemic status. This classification system (GRFP or GRFN) was based on text analysis of the language used by the authors of each study in the way they interpreted their results (eg, lower sensitivity, lower specificity, more misdiagnoses). This allowed for standardization of labeling findings from different study designs. GRFP was assigned if studies reported 1) higher HbA_1c_ values in African descent participants compared with other ethnic groups (eg, White participants) at the same glycemic level; 2) lower sensitivity because of less true positives; or 3) lower specificity because of more false positives. GRFN was assigned if studies reported 1) lower HbA_1c_ values in participants compared with other ethnic groups at same glycemic level; 2) lower sensitivity because of more false negatives; or 3) or lower specificity because of less true negatives. Discrepancies in the review process and data extraction were resolved with input from a third author (M.H.R.).

Included studies were grouped based on study population (African American, Afro-Caribbean, and African) and then organized in alphabetical order by the first author’s last name. Studies were labeled numerically as 1 through 12 based on this ordering.

## Results

Of the 12 articles that met the inclusion criteria, studies numbered 1 through 7 analyzed HbA_1c_ performance among African American people (26–32), study number 8 analyzed HbA_1c_ performance among Afro-Caribbean people (33), and studies numbered 9 through12 analyzed HbA_1c_ performance among African people (34–37). All studies were conducted with people living in the United States (Table 1).

**Table 1:** Study Characteristics for Articles Reporting on Glycated Hemoglobin A_1c_ (HbA_1c_) Performance Among African Descent Populations Living in the United States, 2010–2019

Study	First Author (Year); Study	N; Sex	Race/Ethnicity^a^ (%)	Age, y	Location	Study Design	HbA_1c_ Laboratory Analysis Method
**African American**							
1	Bleyer (2010) (26)	N = 885; 43.2% male and 56.8% female	43.5% African American; 56.5% White	≥18	Winston-Salem, North Carolina	Clinical; retrospective study	Cation-exchange column chromatography on an automated HPLC instrument (Variant II Turbo, Bio-Rad Laboratories).
2	Carson (2016); CARDIA study (27)	N = 2,692; 45.5% male and 54.5% female	44% African American; 56% White	Mean (SD): 45.3 (3.6)	Minneapolis, Minnesota; Chicago, Illinois; Birmingham, Alabama; Oakland, California	Database analysis	Whole blood aliquot by ion-exchange HPLC using a Tosoh G7 (Tosoh Bioscience).
3	Cutrona (2015); FACHS (28)	N = 312; 100% female	100% African American	26–92; Mean (SD): 47 (7)	Ames, Iowa; Athens, Georgia	Database analysis	Whole blood aliquot by turbidimetric immunoinhibition (University of Iowa Clinical Pathology Laboratories).
4	Getaneh (2011); NHANES III and DIAMOND Study (29)	N = 16,056^b^; 48.1% male and 51.9% female	4.3% Dominican; 28.9% Hispanic; 26.9% African American; 39.9% White	Range of mean ages: 38.2–63.3	NHANES III: United States. DIAMOND: New York, New York	Database analysis	Diamat HPLC from Bio-Rad Laboratories.^c^
5	Hivert (2019); DPP (30)	N = 2,658; 33% male and 67% female	55.5% White; 20.2% African American; 17.0% Hispanic; 4.4% Asian; 2.9% American Indian	≥25; Mean (SD): 50.7 (10.7)	27 US clinical centers^d^	Clinical	Ion-exchange HPLC instrument (Variant; Bio-Rad Laboratories).
6	Homko (2012) (31)	N = 83; 7.2% male and 92.8% female	100% African American	Mean (SD): 53 (10.4)	Philadelphia, Pennsylvania	Clinical	CDC-approved automated point-of-care analyzer (DCA 2000, Bayer Corporation): monoclonal antibody recognizes glycated N terminus of β chain of hemoglobin.
7	Meigs (2014); BACH Prediabetes Study (32)	N = 1,387; 37.4% male and 62.6% female	27.3% African American; 29.6% Hispanic; 43.0% White	34–87	Boston, Massachusetts	Clinical	Tina-Quant HbA_1c_ generation 2 assay with analytic measurement range of 3.4%–18% (Quest Diagnostics).
**Afro-Caribbean**							
8	Exebio (2012) (33)	N = 128^e^	100% Haitian American	≥35	Miami, Florida	Clinical	Whole blood with close tube sampling, in duplicate (coefficient of variation <1.7%), with Roche Tina Quant Second Generation A1c immunoassay method of Laboratory Corporation of America.
**African**							
9	Briker (2019); The AIA Study (34)	N = 430; 65% male and 35% female	100% African immigrants in the United States	Mean (SD): 38 (10)	Bethesda, Maryland	Clinical	NGSP-certified instruments: BioRad Laboratories Classic Variant (n = 32), Bio-Rad Laboratories Variant II (n = 158), and BioRad Laboratories D10 (n = 240) used sequentially by the NIH Clinical Center for HPLC.
10	Sumner 1 (2015); The AIA Study (35)	N = 216; 68% male and 32% female	100% African immigrants in the United States	20–64; mean (SD): 37 (10)	Bethesda, Maryland	Clinical	NGSP-certified instruments: Classic Variant, Variant II, and D10 for HPLC (Bio-Rad Laboratories). Whole blood samples in 90 participants analyzed by boronate affinity chromatography method on NGSP-certified Premier Hb9210 analyzer (Trinity Biotech).
11	Sumner 2 (2016); The AIA Study (36)	N = 236; 69% male and 31% female	100% African immigrants in the United States	20–64; Mean (SD): 39 (10)	Bethesda, Maryland	Clinical	NGSP-certified instruments: Variant II and D10 for HPLC (Bio-Rad Laboratories).
12	Sumner 3 (2016); The AIA Study (37)	N = 217; 69% male and 31% female	100% African immigrants in the United States	20–64; Mean (SD): 39 (10)	Bethesda, Maryland	Clinical	NGSP-certified instruments: Variant II and D10 for HPLC (Bio-Rad Laboratories).

Abbreviations: AIA, Africans in America; BACH, Boston Area Community Health; CARDIA, Coronary Artery Risk Development in Young Adults; CDC, Centers for Disease Control and Prevention; DIAMOND, Diabetes Among Dominicans and Other Minorities in Northern Manhattan; DPP, Diabetes Prevention Program; FACHS, Family and Community Health Study; HPLC, high performance liquid chromatography; NHANES III, the third National Health and Nutrition Examination Survey; NIH, National Institutes of Health; NGSP, National Glycohemoglobin Standardization Program.

a For all studies, White refers to Caucasian, Non-Hispanic White, and/or European White.

b Participant data extracted from Table 1, “Sociodemographic Characteristics of Dominicans and the Third National Health and Nutrition Examination Survey Populations, Stratified by Hemoglobin A_1c_-Based Diabetes Diagnosis” (29).

c Laboratory analysis data extracted from “Plan and Operation of the Third National Health and Nutrition Examination Survey, 1988–94. Series 1: Programs and Collection Procedures” (38).

d Location data extracted from “The Diabetes Prevention Program. Design and methods for a clinical trial in the prevention of type 2 diabetes” (39).

e Breakdown for sex/gender not available.

The population size of the studies varied from 83 to 16,056 participants, with the sex representation ranging from 69% male/31% female to 0% male/100% female (Table 1). The study cohorts consisted of 20.2% to 100% African descent populations. The overall age range across the different studies was 18 to 92 years and the mean age was between 37 and 64 years when reported (Table 1).

HbA_1c_ laboratory analysis methods were high performance liquid chromatography (HPLC) for studies 1, 2, 4, 5, and 9 through 12 (26,27,29,30,34–37), or immunoassays for studies 3 and 6 through 8 (28,31–33) (Table 1).

The study designs included either clinical data collection (studies 1 and 5 through 12) (26,30–37) or analyses of established databases (studies 2 through 4) (27–29), with publication dates ranging from 2010 to 2019 (Table 1). Study 1 was a retrospective study of patients who underwent HbA_1c_ testing from May 2008 to February 2009 (Table 1) (26). Study 2 was a cross cross-sectional analysis within the longitudinal Coronary Artery Risk Development in Young Adults (CARDIA) study (Table 1) (27).

In these studies, HbA_1c_ performance was evaluated by comparing HbA_1c_ results in African descent populations to HbA_1c_ results in other ethnic groups (eg, White people) (studies 1, 2, 5, and 7) (26,27,30,32), evaluating HbA_1c_ test results against the 2-hour OGTT, FPG, glycated plasma proteins test results, or previous diagnosis in the same participants (studies 3, 6, and 8–12) (28,31,33–37), or both (study 4) (29) (Table 2). Studies conducted among African American people showed that the HbA_1c_ test almost always had a GRFP in this population. Studies 1, 2, 4, 5, and 7 demonstrated that HbA_1c_ values were higher in African Americans when compared with Whites across a range of glycemic states (26,27,29,30,32). Additionally, Study 7 showed that HbA_1c_ values were higher in African American people when compared with both White people and Hispanic people, leading to the potential of overdiagnosis of type 2 diabetes in African American people (32). Using OGTT as a standard test for diagnosis of glycemic status, studies 4 and 6 demonstrated that using HbA_1c_ results in overdiagnosis of type 2 diabetes when HbA_1c_ is 6.5% or higher (29,31). Study 3 showed that African American people may experience an overdiagnosis of prediabetes or type 2 diabetes at HbA_1c_ of 5.7% to less than 6.5%; however, study 6 showed that an HbA_1c_ cutoff of less than 5.7% does not eliminate the possibility of a type 2 diabetes diagnosis (28,31) (Table 2).

**Table 2:** Evaluation of Glycated Hemoglobin A_1c_ (HbA_1c_) Performance: Greater Risk of False Positives Versus Greater Risk of False Negatives Among African Descent Populations Living in the United States, 2010–2019

Study	HbA_1c_ Evaluation Method	Findings	Performance
**African American**			
1	Compared with other ethnic groups (ie, White people)	Main finding: Higher HbA_1c_ values for African American than for White people at all fasting glucose levels (26).	Greater risk of false positives
		Additional findings: Relationship between HbA_1c_ and simultaneous serum glucose did not differ between African American people with and without the SCT. SCT does not impact relationship between HbA_1c_ and serum glucose concentration, and does not account for differences between African American and White people.	
2	Compared with other ethnic groups (ie, White people)	Main finding: African American people without previous diagnosis of type 2 diabetes by OGTT had higher mean values of HbA_1c_ than White people (β = 0.19% points; 95% CI = 0.14–0.24) (27).	Greater risk of false positives
		Additional finding: HbA_1c_ values were compared for participants free of type 2 diabetes based on the OGTT.	
3	Compared with other measures (ie, previous diagnosis)^a^	Main finding: Chronic financial strain increased sIL-6R, an inflammatory marker, and HbA_1c_ (28).	Greater risk of false positives
		Additional finding: Although African American women had no previous prediabetes or type 2 diabetes diagnosis, 54% had HbA_1c_ >5.7%.	
4	Compared with other ethnic groups (ie, White people); Compared with other measures (ie, FPG and OGTT)	Main findings: For African American people (N = 408) classified as having normal glucose tolerance by either FPG or OGTT, HbA_1c_ misclassified 3.5% of them as having type 2 diabetes (29). HbA_1c_ diagnosed type 2 diabetes in 67% of African American people and 37.9% of White people.	Greater risk of false positives
5	Compared with other ethnic groups (ie, White people)	Main finding: HbA_1c_ was higher in African American (mean [SD], 6.2% [0.6]) than in White people (mean [SD], 5.8% [0.4]) (30).	Greater risk of false positives
		Additional findings: Genomic analysis showed that 3 genetic factors contributed to the differences in HbA_1c_: PCA factor, SCT, and GRS. 60% of HbA_1c_ differences between African American and White people are explained by first genomic PCA factor (degree of African ancestry). SCT explained 16% of the difference and GRS explained 14% of difference in HbA_1c_ between African American and White people.	
6	Compared with other measures (ie, OGTT)	Main findings: For patients with type 2 diabetes diagnosis by HbA_1c_, OGTT classified 48.3% with type 2 diabetes, 38.7% with IGT, and 12.9% with normal glucose tolerance. HbA_1c_ ≤5.6% does not exclude type 2 diabetes or IGT. Among 33.7% of patients with HbA_1c_ ≤5.6%, 64.3% had IGT or type 2 diabetes (31).	Greater risk of false positives at HbA_1c_ ≥6.5% and greater risk of false negatives at HBA_1c_ ≤5.6%
		Additional findings: 15.9% of patients had HbA_1c_ ≥6.5%. HbA_1c_ ≥6.5% indicates type 2 diabetes or IGT, with 50% sensitivity and 90% specificity. HbA_1c_ ≥6.5% had positive predictive value of 48%. HbA_1c_ ≤5.6% showed 17.2% sensitivity and 100% specificity.	
7	Compared with other ethnic groups (ie, Hispanic and White people)	Main finding: Mean HbA_1c_ levels were higher in African American (5.68%) than in Hispanic (5.57%) and White people (5.47%) (32).	Greater risk of false positives
		Additional findings: With every 1% increase in European ancestry, there was a 0.002% decrease in HbA_1c_. Individuals with 100% African American ancestry had an HbA_1c_ value that was 0.27% higher than those with 100% European ancestry.	
**Afro-Caribbean**			
8	Compared with other measures (ie, FPG)	Main findings: At HbA_1c_ ≥6.5%, sensitivity was 73% and specificity was 89%. At HbA_1c_ ≥6.26%, sensitivity was 80% and specificity was 74% (33).	Greater risk of false negatives
		Additional finding: The area under the ROC curve for HbA_1c_ as a diagnostic indicator of type 2 diabetes was 0.86.	
**African**			
9	Compared with other measures (ie, OGTT)	Main findings: For 32 individuals with type 2 diabetes, HbA_1c_ detected type 2 diabetes in 32% and OGTT detected type 2 diabetes in 68% of individuals with HbA_1c_ <6.5%. For 178 individuals with prediabetes, HbA_1c_ detected prediabetes in 57% of individuals and OGTT detected prediabetes in 43% of individuals.	Greater risk of false negatives
		Additional finding: Using HbA_1c_ alone missed a diagnosis of type 2 diabetes in 60% of African people and a prediabetes diagnosis in 40% of African people (34).	
10	Compared with other measures (ie, FPG and OGTT)	Main finding: Among subjects with IGT by OGTT, HbA_1c_ ≥5.7% had sensitivity of 53%, 54%, and 47% for the total, normal, and variant hemoglobin groups, respectively (35).	Greater risk of false negatives
		Additional findings: HbA_1c_ with FPG demonstrated sensitivity of 64%. HbA_1c_ diagnostic sensitivity did not vary by variant hemoglobin status.	
11	Compared with other measures (ie, OGTT and glycated albumin)	Main finding: Among subjects with prediabetes by OGTT, HbA_1c_ of 5.7% to less than 6.5% had 37% sensitivity in nonobese African immigrants and 64% sensitivity in obese African immigrants (36).	Greater risk of false negatives
		Additional finding: For HbA_1c_ of 5.7% to less than 6.5% combined with glycated albumin ≥13.77%, sensitivity increased to 72% for nonobese African immigrants.	
12	Compared with other measures (ie, OGTT and glycated albumin)	Main findings: When type 2 diabetes was detected by glycated plasma proteins (albumin or fructosamine; n = 24), average HbA_1c_ was mean (SD) 5.2% (0.4). OGTT detected prediabetes in 74 individuals (13 of 74 had low HbA_1c_) (37).	Greater risk of false negatives
		Additional findings: HbA_1c_ detected ≤50% of African immigrants with prediabetes. HbA_1c_ combined with the glycated albumin test increases sensitivity to 80% for diagnosing prediabetes.	

Abbreviations: OGTT, 2-hour oral glucose tolerance test; FPG, fasting plasma glucose; IGT, impaired glucose tolerance; PCA, principal component analysis; GRS, genetic risk score; SCT, sickle cell trait; ROC, receiver operating characteristic.

a Exact temporality between the previous diagnosis and HbA_1c_ testing was not provided within the study, with an estimate of less than 12 months extrapolated from the study design. Findings from this study may represent new onset diabetes. This provides a limitation in the conclusive findings for HbA_1c_ performance in this study.

In the Afro-Caribbean population, HbA_1c_ testing at the 6.5% or higher cutoff has a GRFN (33). Using FPG as a standard for diagnosis of type 2 diabetes, study 8 showed that more participants were correctly diagnosed as having type 2 diabetes if the cutoff was lowered to 6.26% or higher, suggesting that HbA_1c_ values are generally lower in Afro-Caribbean people (Table 2).

The Africans in America studies 9 through 12 all showed that HbA_1c_ has a GRFN in African people at the HbA_1c_ cutoff of 5.7% to less than 6.5% for prediabetes and HbA_1c_ cutoff of 6.5% or higher for type 2 diabetes (34–37). Using OGTT as a diagnostic standard for glycemic status, studies 9 through 12 demonstrated that using an HbA_1c_ cutoff of 5.7% to less than 6.5% will lead to underdiagnosis of prediabetes in Africans. Additionally, study 9 showed that using an HbA_1c_ cutoff of 6.5% or higher will lead to an underdiagnosis of type 2 diabetes in Africans (34) (Table 2).

## Discussion

We assessed 12 studies that evaluated the ability of HbA_1c_ to correctly identify African American, Afro-Caribbean, and African people with prediabetes or type 2 diabetes. Studies among African American people found that HbA_1c_ of 5.7% to less than 6.5% or HbA_1c_ of 6.5% or higher led to overdiagnosis. In one study of Afro-Caribbean people, HbA_1c_ of 6.5% or higher had a greater risk of false negatives (GRFN). Among African people, HbA_1c_ of 5.7% to less than 6.5% or HbA_1c_ of 6.5% or higher led to greater risk of underdiagnosis.

Overdiagnosis of diabetes was likely among African American people in 3 ways. African American people had consistently higher HbA_1c_ levels than White people regardless of glycemic status (26,27,29,30,32). Furthermore, half of normoglycemic African American people had HbA_1c_ values greater than 5.7% (28); and lastly, African American people were more likely to be diagnosed with type 2 diabetes by HbA_1c_ of 6.5% or higher alone but not by OGTT (29,31). Although study 6 did suggest a GRFN at HbA_1c_ less than 5.7%, by misdiagnosing some participants as having normal glycemic status if their HbA_1c_ was less than 5.7% (31), the finding is limited by the smaller sample size of 83 participants when compared with the other studies. This finding must be investigated further.

In Afro-Caribbean people, the HbA_1c_ cutoff of 6.5% is likely to result in underdiagnosis of type 2 diabetes because study 8 showed that more participants were correctly diagnosed as having type 2 diabetes if the cutoff was lowered to 6.26% (33). However, this finding may not be generalizable to other Afro-Caribbean populations because of the smaller sample size and limitation of the study population to Haitian American people. Additionally, because only 1 study provided this conclusion, generalizability is further limited. For African people, underdiagnosis of prediabetes and type 2 diabetes is also likely at the standard HbA_1c_ cutoffs because diagnosis was missed by HbA_1c_ despite being detected by OGTT (34–37). The findings among African people hold true regardless of hemoglobin variant or obesity status (35,36).

Genetics are often thought to be responsible for the differences of HbA_1c_ performance in African descent populations (24,40–43). In fact, genetic analysis in study 5 shows that the HbA_1c_ difference was primarily because of the genomic principal component analysis (PCA) factor in African American people when compared with White people (30). The study demonstrated that the PCA factor was associated with increased HbA_1c_ values in African American people. However, genetics do not fully explain HbA_1c_ differences among African American people (44), because increases in HbA_1c_ may be mediated by social determinants of health (eg, chronic financial strain as seen in study 3) or chronic inflammation (sIL-6R) (28,45). Additionally, G6PD variant or deficiency is often correlated with lower HbA_1c_ values in various populations (40), especially in African American people and African people because of its higher prevalence in these groups (14,46,47). Similarly, the sickle cell trait is associated with lower HbA_1c_ values in African descent populations (21,25). However, study 1 showed that the sickle cell trait may not actually correlate to changes in HbA_1c_ values for African American people (26). Findings regarding associations of genetics with HbA_1c_ are still being researched in this population. Research accounting for genetically linked HbA_1c_ differences in Afro-Caribbean people is also lacking. Genetic polymorphisms between African American people and Haitian people have been researched and show that differences in the *PPARGC1A* gene will correlate to risk of type 2 diabetes in African American people as opposed to protective associations with type 2 diabetes in Haitian people, suggesting that other genetic associations may explain differences in diabetes for Haitian people (48). Although little research explains the role of genetics in HbA_1c_ differences for Haitian people, one likely contributor to lower HbA_1c_ values may be the G6PD variant because of its higher prevalence in populations of African descent (47). Nevertheless, opposing findings regarding the role of genetics in influencing HbA_1c_ values (eg, PCA factor is associated with higher HbA_1c_ whereas the sickle cell trait is associated with lower HbA_1c_) make it difficult to ascertain the overall impact genetics has in causing the differences in HbA_1c_ that were found for the African descent populations and therefore require further evaluation.

Socioeconomic factors and health behaviors such as diet, smoking, and exercise may explain some differences in glycemic control and HbA_1c_ values among the 3 groups. Higher income and educational attainment appear to decrease the odds of diabetes among African immigrants, whereas only higher education lowers the odds for African American people (5). Neither education nor income appear to affect diabetes risk among Afro-Caribbean people (5,49). Additionally, study 3 found that financial stress and chronic inflammation were associated with higher HbA_1c_. Chronic inflammation resulting from social and environmental stressors, including experiences of racism, correlate to higher HbA_1c_ in nondiabetic adults (50). In terms of health behaviors, compared with African American people, African and Afro-Caribbean people are less likely to smoke. As African and Afro-Caribbean immigrants settle in the United States, they are affected by dietary acculturation often characterized by increased caloric intake and diets higher in refined carbohydrates, animal protein, fat, and sodium (5). Although diet may affect glycemic control, it is unlikely that diet explains the differences in HbA_1c_ performance illustrated in this study. These socioeconomic factors highlight the diversity of experience within African descent groups, which is often overshadowed by perceived homogeneity of the “Black” experience in the United States. Since immigration to the United States presents unique socioeconomic circumstances that can affect factors like HbA_1c_ (4), impacts of these circumstances are important to analyze distinctly from global concerns.

With these factors affecting HbA_1c_ performance, results must be interpreted with caution. Some alternative diagnostic tests are suggested to aid or replace HbA_1c_ for classification of glycemic status. For example, FPG in combination with HbA_1c_ increases the sensitivity for type 2 diabetes diagnosis in African people (study 10) (35). A stronger relationship between HbA_1c_ and FPG at higher FPG levels in most ethnic groups has been suggested as well (51). Study 8 suggests that FPG may be a better measure of glycemic status than HbA_1c_ in Afro-Caribbean people (33). At the same time, studies 3, 6, and 9 through 12 suggest that OGTT more accurately measures glycemic status than HbA_1c_ in both African American and African people (28,31,34–37). Comparisons between HbA_1c_ and OGTT in Afro-Caribbean people are lacking and should be studied further.

Convenient nonfasting alternatives for type 2 diabetes testing are other glycated proteins (eg, glycated albumin, fructosamine, and other advanced glycation end products) either in combination with or in place of HbA_1c_ (36,37,52–55). Although this approach is supported in multiethnic studies, these glycated proteins should be evaluated specifically in African descent groups.

Several limitations exist for the findings of our review. Despite constructing a comprehensive search, articles published in peer reviewed journals that were not indexed in PubMed, Scopus, and CINAHL may have been missed. The search contained nouns and adjectives as identification for African descent countries and regions of origin and HbA_1c_ testing. However, study participant groups may be based on self or researcher categorization rather than actual region, country, or ethnic group of the participant. Findings must be interpreted with caution because of this subjective labeling within studies. Additionally, we did not use a specific protocol to evaluate the quality of the included studies, as this is not a part of scoping review methodologies and can increase risk of bias (56,57). Another limitation that must be considered is that time may pass between HbA_1c_ testing and alternate testing in some studies and glycemic status of individuals can change in that time; this limitation will usually exist in this nature of clinical research methodology and therefore must be recognized when evaluating the conclusions from those studies.

According to our review process, there is only 1 study protocol in the United States that examines performance of diabetes screening tests among African immigrants to the United States (34–37). However, studies 9 through 12 demonstrate distinct comparisons within this cohort that illustrate significant conclusions about HbA_1c_ performance. This is because the protocol is ongoing, and the number of participants increased over time. In turn, this also lends strength to the findings, because the similarity in protocol is balanced by the increasing diversity of the sample for each study design.

Finally, the lack of existing studies for Afro-Caribbean people in the United States presents a substantial limitation; our findings for this group must be interpreted cautiously. Further research is needed to understand the performance of HbA_1c_ and evaluate alternate tests in place of the HbA_1c_ in specific African descent populations, especially Afro-Caribbean people. Unique settings like New York City, where 32% of the African descent population is Afro-Caribbean and 4% is African (58), may serve as key locations for public health researchers to investigate type 2 diabetes screening and diagnostics.

Our review also has several strengths. In partnership with our institution’s research librarian, we tested several search constructions and selected the searches that provided the broadest selection within the scope of our topic. Additionally, we searched 3 databases without limiting article type or study designs on title and abstract review and had 2 reviewers independently screen the articles. This improved the selection of articles available for review and reduced selection bias. Finally, we were able to provide clear findings by constructing a label categorization scheme (GRFP/GRFN) that allowed for grouping of studies that used different comparative analytic and statistical methods to analyze HbA_1c_.

In African descent populations in the United States, the utility of HbA_1c_ is limited in screening for glycemic status, determining care methods, assessing risk of type 2 diabetes complications, or analyzing health disparities. Current HbA_1c_ cutoffs for prediabetes and type 2 diabetes may overestimate glycemic status in African American people and underestimate glycemic status in Afro-Caribbean and African people. Reasons for variations in HbA_1c_ have been attributed to genetic, biochemical, and socioeconomic factors. Alternate testing such as OGTT, FPG, and other glycated blood proteins in place of or in combination with HbA_1c_ may better assess glycemic status in African descent populations. Intraethnic HbA_1c_ heterogeneity within the African descent groups must be recognized, and identification of more reliable type 2 diabetes screening and diagnostic tests is urgent.

## Appendix Search Strings Used in a Scoping Review of HbA_1c_ Performance in African Descent Populations in the United States With Normal Glucose Tolerance, Prediabetes, and Diabetes

**Table:**

Database	Search String
PubMed	(africa[tiab] OR african[tiab] OR africans[tiab] OR “africa”[MeSH Terms] OR afro[tiab] OR black[tiab] OR “african continental ancestry group”[MeSH Terms] OR “african americans”[MeSH Terms] OR Angola[tiab] OR Angolan[tiab] OR Benin[tiab] OR Beninese[tiab] OR Botswana[tiab] OR Motswana[tiab] OR Batswana[tiab] OR “Burkina Faso”[tiab] OR Burkinabe[tiab] OR Burundi[tiab] OR Burundian[tiab] OR Cameroon[tiab] OR Cameroonian[tiab] OR “Cape Verde”[tiab] OR “Cape Verdean”[tiab] OR “Central African Republic”[tiab] OR “Central African”[tiab] OR Chad[tiab] OR Chadian[tiab] OR Comoros[tiab] OR Comorian[tiab] OR “Republic of the Congo”[tiab] OR Congolese[tiab] OR Djibouti[tiab] OR Djiboutian[tiab] OR “Equatorial Guinea”[tiab] OR “Equatorial Guinean”[tiab] OR Equatoguinean[tiab] OR Eritrea[tiab] OR Eritrean[tiab] OR Ethiopia[tiab] OR Ethiopian[tiab] OR Gabon[tiab] OR Gabonese[tiab] OR Gambia[tiab] OR Gambian[tiab] OR Ghana[tiab] OR Ghanaian[tiab] OR Guinea[tiab] OR Guinean[tiab] OR “Guinea-Bissau”[tiab] OR “Bissau-Guinean”[tiab] OR “Ivory Coast”[tiab] OR Ivorian[tiab] OR Kenya[tiab] OR Kenyan[tiab] OR Lesotho[tiab] OR Mosotho[tiab] OR Basotho[tiab] OR Liberia[tiab] OR Liberian[tiab] OR Madagascar[tiab] OR Malagasy[tiab] OR Malawi[tiab] OR Malawian[tiab] OR Mali[tiab] OR Malian[tiab] OR Mauritania[tiab] OR Mauritanian[tiab] OR Mauritius[tiab] OR Mauritian[tiab] OR Mozambique[tiab] OR Mozambican[tiab] OR Namibia[tiab] OR Namibian[tiab] OR Niger[tiab] OR Nigerien[tiab] OR Nigeria[tiab] OR Nigerian[tiab] OR Rwanda[tiab] OR Rwandan[tiab] OR “Sao Tome and Principe”[tiab] OR Senegal[tiab] OR Senegalese[tiab] OR Seychelles[tiab] OR Seychellois[tiab] OR “Sierra Leone”[tiab] OR “Sierra Leonean”[tiab] OR Somalia[tiab] OR Somalian[tiab] OR “South Africa”[tiab] OR “South African”[tiab] OR “South Sudan”[tiab] OR “South Sudanese”[tiab] OR Sudan[tiab] OR Sudanese[tiab] OR Swaziland[tiab] OR Swazi[tiab] OR Tanzania[tiab] OR Tanzanian[tiab] OR Togo[tiab] OR Uganda[tiab] OR Ugandan[tiab] OR Zambia[tiab] OR Zambian[tiab] OR Zimbabwe[tiab] OR Zimbabwean[tiab] OR anguilla[tiab] OR anguillian[tiab] OR “Antigua and Barbuda”[tiab] OR antiguan[tiab] OR barbudan[tiab] OR aruba[tiab] OR aruban[tiab] OR bahamas[tiab] OR bahamian[tiab] OR barbados[tiab] OR barbadian[tiab] OR belize[tiab] OR belizean[tiab] OR bermuda[tiab] OR bermudian[tiab] OR “British Virgin Islands”[tiab] OR caribbean[tiab] OR “Cayman Islands”[tiab] OR “Costa Rica”[tiab] OR “Costa Rican”[tiab] OR cuba[tiab] OR cuban[tiab] OR curacao[tiab] OR curacaoans[tiab] OR dominica[tiab] OR “Dominican Republic”[tiab] OR dominican[tiab] OR grenada[tiab] OR grenadine[tiab] OR guadeloupe[tiab] OR guadeloupean[tiab] OR guyana[tiab] OR guyanese[tiab] OR haiti[tiab] OR haitian[tiab] OR honduras[tiab] OR honduran[tiab] OR jamaica[tiab] OR jamaican[tiab] OR martinique[tiab] OR martiniquais[tiab] OR montserrat[tiab] OR montserratian[tiab] OR nevis[tiab] OR nicaragua[tiab] OR nicaraguan[tiab] OR panama[tiab] OR panamanian[tiab] OR “Puerto Rico”[tiab] OR “Puerto Rican”[tiab] OR “St. Barts”[tiab] OR “St. Christopher”[tiab] OR “St. Croix”[tiab] OR “St. Johns”[tiab] OR “St. Kitts and Nevis”[tiab] OR “St. Lucia”[tiab] OR “St. Martin”[tiab] OR “St. Thomas”[tiab] OR “St. Vincent”[tiab] OR vincentian[tiab] OR suriname[tiab] OR surinamese[tiab] OR “Trinidad and Tobago”[tiab] OR trinidadian[tiab] OR trini[tiab] OR tobagonian[tiab] OR “US Virgin Islands”[tiab] OR venezuela[tiab] OR venezuelan[tiab] OR “Virgin Islands”[tiab] OR “West Indies”[tiab] OR “West Indian”[tiab]) AND (“Glycated Hemoglobin A”[mesh] OR “hemoglobin A1c”[tiab] OR “hba1c”[tiab] OR “A1C”[tiab]) AND (“2000/01/01”[PDAT]: “2020/01/01”[PDAT])
Scopus	(TITLE-ABS-KEY ((africa OR african OR africans OR afro OR black OR “african americans” OR blacks OR angola OR angolan OR benin OR beninese OR botswana OR motswana OR batswana OR “Burkina Faso” OR burkinabe OR burundi OR burundian OR cameroon OR cameroonian OR “Cape Verde” OR “Cape Verdean” OR “Central African Republic” OR “Central African” OR chad OR chadian OR comoros OR comorian OR “Republic of the Congo” OR congolese OR djibouti OR djiboutian OR “Equatorial Guinea” OR “Equatorial Guinean” OR equatoguinean OR eritrea OR eritrean OR ethiopia OR ethiopian OR gabon OR gabonese OR gambia OR gambian OR ghana OR ghanaian OR guinea OR guinean OR “Guinea-Bissau” OR “Bissau-Guinean” OR “Ivory Coast” OR ivorian OR kenya OR kenyan OR lesotho OR mosotho OR basotho OR liberia OR liberian OR madagascar OR malagasy OR malawi OR malawian OR mali OR malian OR mauritania OR mauritanian OR mauritius OR mauritian OR mozambique OR mozambican OR namibia OR namibian OR niger OR nigerien OR nigeria OR nigerian OR rwanda OR rwandan OR “Sao Tome and Principe” OR senegal OR senegalese OR seychelles OR seychellois OR “Sierra Leone” OR “Sierra Leonean” OR somalia OR somalian OR “South Africa” OR “South African” OR “South Sudan” OR “South Sudanese” OR sudan OR sudanese OR swaziland OR swazi OR tanzania OR tanzanian OR togo OR uganda OR ugandan OR zambia OR zambian OR zimbabwe OR zimbabwean OR anguilla OR anguillian OR “Antigua and Barbuda” OR antiguan OR barbudan OR aruba OR aruban OR bahamas OR bahamian OR barbados OR barbadian OR belize OR belizean OR bermuda OR bermudian OR “British Virgin Islands” OR caribbean OR “Cayman Islands” OR “Costa Rica” OR “Costa Rican” OR cuba OR cuban OR curacao OR curacaoans OR dominica OR “Dominican Republic” OR dominican OR grenada OR grenadine OR guadeloupe OR guadeloupean OR guyana OR guyanese OR haiti OR haitian OR honduras OR honduran OR jamaica OR jamaican OR martinique OR martiniquais OR montserrat OR montserratian OR nevis OR nicaragua OR nicaraguan OR panama OR panamanian OR “Puerto Rico” OR “Puerto Rican” OR “St. Barts” OR “St. Christopher” OR “St. Croix” OR “St. Johns” OR “St. Kitts and Nevis” OR “St. Lucia” OR “St. Martin” OR “St. Thomas” OR “St. Vincent” OR vincentian OR suriname OR surinamese OR “Trinidad and Tobago” OR trinidadian OR trini OR tobagonian OR “US Virgin Islands” OR venezuela OR venezuelan OR “Virgin Islands” OR “West Indies” OR “West Indian”)) AND DOCTYPE (ar OR re) AND PUBYEAR > 1999) AND (TITLE-ABS-KEY ((hba1c OR “glycosylated hemoglobin A” OR “glycated hemoglobin” OR “hemoglobin A1c” OR “glycated hemoglobin A”))) AND (LIMIT-TO (SUBJAREA, “MEDI”) OR LIMIT-TO (SUBJAREA, “NURS”) OR LIMIT-TO (SUBJAREA, “HEAL”) OR LIMIT-TO (SUBJAREA, “SOCI”)) AND (LIMIT-TO (LANGUAGE, “English”))
Cumulative Index to Nursing and Allied Health Literature (CINAHL)	(hba1c OR glycosylated hemoglobin A OR glycated hemoglobin OR “hemoglobin A1c” OR “glycated hemoglobin A”) AND (africa OR african OR africans OR afro OR black OR african americans OR blacks OR caribbean OR Angola OR Angolan OR Benin OR Beninese OR Botswana OR Motswana OR Batswana OR “Burkina Faso” OR Burkinabe OR Burundi OR Burundian OR Cameroon OR Cameroonian OR “Cape Verde” OR “Cape Verdean” OR “Central African Republic” OR “Central African” OR Chad OR Chadian OR Comoros OR Comorian OR “Republic of the Congo” OR Congolese OR Djibouti OR Djiboutian OR “Equatorial Guinea” OR “Equatorial Guinean” OR Equatoguinean OR Eritrea OR Eritrean OR Ethiopia OR Ethiopian OR Gabon OR Gabonese OR Gambia OR Gambian OR Ghana OR Ghanaian OR Guinea OR Guinean OR “Guinea-Bissau” OR “Bissau-Guinean” OR “Ivory Coast” OR Ivorian OR Kenya OR Kenyan OR Lesotho OR Mosotho OR Basotho OR Liberia OR Liberian OR Madagascar OR Malagasy OR Malawi OR Malawian OR Mali OR Malian OR Mauritania OR Mauritanian OR Mauritius OR Mauritian OR Mozambique OR Mozambican OR Namibia OR Namibian OR Niger OR Nigerien OR Nigeria OR Nigerian OR Rwanda OR Rwandan OR “Sao Tome and Principe” OR Senegal OR Senegalese OR Seychelles OR Seychellois OR “Sierra Leone” OR “Sierra Leonean” OR Somalia OR Somalian OR “South Africa” OR “South African” OR “South Sudan” OR “South Sudanese” OR Sudan OR Sudanese OR Swaziland OR Swazi OR Tanzania OR Tanzanian OR Togo OR Uganda OR Ugandan OR Zambia OR Zambian OR Zimbabwe OR Zimbabwean OR anguilla OR anguillian OR “Antigua and Barbuda” OR antiguan OR barbudan OR aruba OR aruban OR bahamas OR bahamian OR barbados OR barbadian OR belize OR belizean OR bermuda OR bermudian OR “British Virgin Islands” OR caribbean OR “Cayman Islands” OR “Costa Rica” OR “Costa Rican” OR cuba OR cuban OR curacao OR curacaoans OR dominica OR “Dominican Republic” OR dominican OR grenada OR grenadine OR guadeloupe OR guadeloupean OR guyana OR guyanese OR haiti OR haitian OR honduras OR honduran OR jamaica OR jamaican OR martinique OR martiniquais OR montserrat OR montserratian OR nevis OR nicaragua OR nicaraguan OR panama OR panamanian OR “Puerto Rico” OR “Puerto Rican” OR “St. Barts” OR “St. Christopher” OR “St. Croix” OR “St. Johns” OR “St. Kitts and Nevis” OR “St. Lucia” OR “St. Martin” OR “St. Thomas” OR “St. Vincent” OR vincentian OR suriname OR surinamese OR “Trinidad and Tobago” OR trinidadian OR trini OR tobagonian OR “US Virgin Islands” OR venezuela OR venezuelan OR “Virgin Islands” OR “West Indies” OR “West Indian”)
